# Biological Evaluation of Double Point Modified Analogues of 1,25-Dihydroxyvitamin D_2_ as Potential Anti-Leukemic Agents

**DOI:** 10.3390/ijms17020091

**Published:** 2016-02-01

**Authors:** Aoife Corcoran, Sharmin Nadkarni, Kaori Yasuda, Toshiyuki Sakaki, Geoffrey Brown, Andrzej Kutner, Ewa Marcinkowska

**Affiliations:** 1 Faculty of Biotechnology, University of Wroclaw, 14a Joliot-Curie, Wroclaw 50-383, Poland; aoifecorkie@hotmail.com; 2 Pharmaceutical Research Institute, 8 Rydygiera, Warsaw 01-793, Poland; s.nadkarni@ifarm.eu (S.N.); a.kutner@ifarm.eu (A.K.); 3 Department of Biotechnology, Faculty of Engineering, Toyama Prefectural University, 5180 Kurokawa, Imizu, Toyama 939-0398, Japan; z02232@mail.pu-toyama.ac.jp (K.Y.); tsakaki@pu-toyama.ac.jp (T.S.); 4 School of Immunity and Infection, University of Birmingham, Vincent Drive, Edgbaston, Birmingham, West Midlands B15 2TT, UK; G.BROWN@bham.ac.uk

**Keywords:** vitamin D analogues, leukemia, receptor

## Abstract

Structurally similar double-point modified analogues of 1,25-dihydroxyvitamin D_2_ (1,25D_2_) were screened *in vitro* for their pro-differentiating activity against the promyeloid cell line HL60. Their affinities towards human full length vitamin D receptor (VDR) and metabolic stability against human vitamin D 24-hydroxylase (CYP24A1) were also tested. The analogues (PRI-1730, PRI-1731, PRI-1732, PRI-1733 and PRI-1734) contained 5,6-*trans* modification of the A-ring and of the triene system, additional hydroxyl or unsaturation at C-22 in the side chain and reversed absolute configuration (24-*epi*) at C-24 of 1,25D_2_. As presented in this paper, introduction of selected structural modifications simultaneously in two distinct parts of the vitamin D molecule resulted in a divergent group of analogues. Analogues showed lower VDR affinity in comparison to that of the parent hormones, 1,25D_2_ and 1,25D_3_, and they caused effective HL60 cell differentiation only at high concentrations of 100 nM and above. Unexpectedly, introducing of a 5,6-*trans* modification combined with C-22 hydroxyl and 24-*epi* configuration switched off entirely the cell differentiation activity of the analogue (PRI-1734). However, this analogue remained a moderate substrate for CYP24A1, as it was metabolized at 22%, compared to 35% for 1,25D_2_. Other analogues from this series were either less (12% for PRI-1731 and PRI-1733) or more (52% for PRI-1732) resistant to the enzymatic deactivation. Although the inactive analogue PRI-1734 failed to show VDR antagonism, when tested in HL60 cells, its structure might be a good starting point for our design of a vitamin D antagonist.

## 1. Introduction

1,25-Dihydroxyvitamin D_3_ (1,25D_3_) belongs to the family of steroid/thyroid hormones. Initially 1,25D_3_ was discovered as a potent anti-rachitic agent [1], but its roles in immunomodulation, cell proliferation and differentiation, as well as cancer prevention were later discovered [2,3]. Vitamin D_3_, a precursor of 1,25D_3_, is produced from 7-dehydrocholesterol in human skin, when exposed to UV-light. In many regions, there is a lack of sunlight in the wintertime, which leads to vitamin D_3_ deficiency. In order to counteract this deficiency, it is necessary to incorporate foods with supplementary vitamin D into the diet. Food from animal sources contains vitamin D_3_, while from plants, vitamin D_2_.

**Figure 1:**
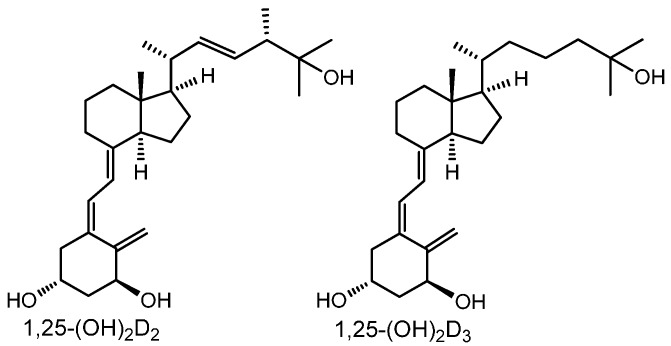
Structures of 1,25D_2_ and 1,25D_3_.

1,25D_2_ differs from 1,25D_3_ by the additional unsaturation at C-22 and a methyl at C-24 (presented in Figure 1); whether these two forms are equivalent when used to prevent rachitis and osteoporosis has been highly debated [4]. Because of their multiple functions, analogues of 1,25D_3_ are currently used to treat psoriasis and secondary hyperparathyroidism associated with a chronic renal disease, and are also in clinical trials against prostate cancer and acute myeloid leukemia [5,6,7]. 1,25D_3_ itself is not suitable for most therapeutic purposes, because of its high potential to mobilise calcium from intestines, kidneys, and bones to the blood serum, which may cause calcification of soft tissues. Therapeutic analogues must therefore be selective in their activities, with reduced calcemic activity and enhanced differentiation-inducing potential. In our laboratories, many new analogues were tested in order to select analogues with the desired properties.

Our previous experiments documented that analogues of 1,25D_2_ with one-carbon unit extended and rigidified (PRI-1906), and additionally homologated at both terminal carbons (PRI-1907) aliphatic side-chain revealed particularly strong differentiation-inducing potential [8,9]. In a later attempt to lower the toxicity of PRI-1907, new 1,25D_2_ analogues were designed, which were modified in two distinct parts of the molecule. These structures combined the optimized side-chains of PRI-1906 and PRI-1907 with the known 19-*nor* modification and, as a result, new analogues PRI-5201 and PRI-5202 were obtained. These analogues had reduced calcaemic activities and general toxicity for mice, and strongly enhanced differentiation-inducing potential, when compared to 1,25D_3_ [10]. 1,25D_3_, 1,25D_2_ and analogues in the target cells bind to the nuclear vitamin D receptor (VDR) [11]. VDR, once bound by the ligand in the cytosol, is transported to the cell nucleus, where it acts as a ligand-activated transcription factor in complex with other regulators of transcription [12]. The biological activities of analogues are regulated at multiple levels and depend on the availability of the given analogue in blood serum, its effective transport to the cells, efficient binding to VDR and rate of degradation to inactive metabolites. CYP24A1, an enzyme located in the inner membrane of mitochondria, is responsible for degradation of the compound to its inactive metabolite, calcitroic acid [13]. Therefore, in our current paper, we describe the biological evaluation that has allowed us to describe the structure-function relationships of our new double point modified analogues of 1,25D_2_.

## 2. Results

### 2.1. Differentiation of HL60 Cells

**Figure 2:**
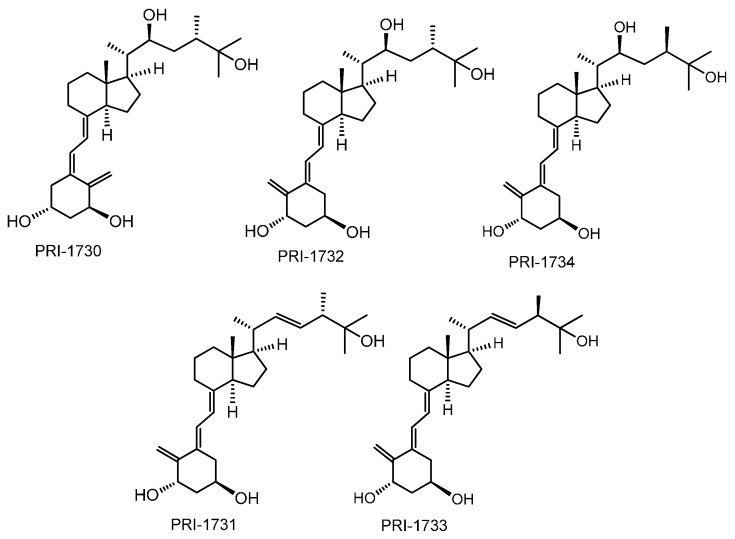
Structures of double point modified analogues of 1,25D_2_ including 22-hydroxy analogues PRI-1730, PRI-1732 and PRI-1734, as well as analogue PRI-1731 with D_2_-like side-chain and its 24-*epi* analogue PRI-1733.

We have previously reported the pro-differentiating activities of double-point modified analogues of 1,25D_2_ [10]. Here, we use the same acute myeloid leukemia (AML) cell line HL60 to examine the pro-differentiating activities of the new analogues: PRI-1730, PRI-1731, PRI-1732, PRI-1733, PRI-1734 [14]. The structures of these analogues are presented in Figure 2.

**Figure 3:**
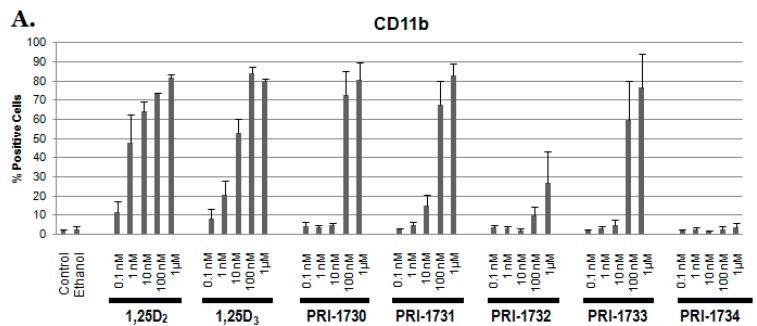

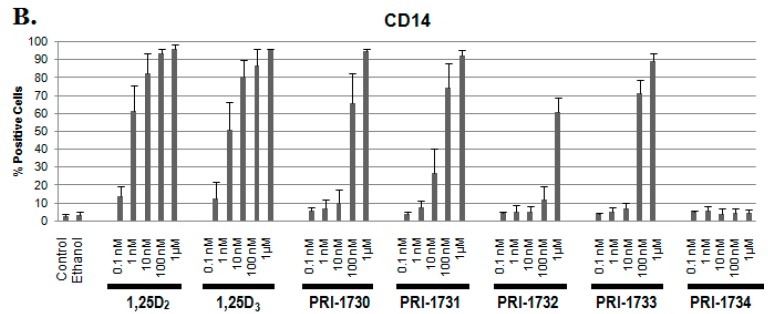
Preliminary screening of pro-differentiating activities of analogues studied. HL60 cells were exposed to 1,25D_2_, 1,25D_3_ and analogues at concentrations of: 0.1, 1, 10, 100 nM, and 1 μM. The expression levels of both (**A**) CD11b and (**B**) CD14 were studied. Percentages of positive cells are presented in the *y*-axis.

Cells were treated with analogues at varying concentrations (0.1, 1, 10, 100 nM and 1 μM) for 96 h after which the expression of the monocyte/macrophage markers CD11b and CD14 were studied using flow cytometry. The data obtained (Figure 3) show that the new analogues have much lower pro-differentiating activities in comparison to that of 1,25D_2_ and 1,25D_3_. In particular, analogue PRI-1734 appears to be completely inactive as there was no upregulation of either CD11b or CD14 at any of the concentrations tested.

### 2.2. Nuclear Translocation of VDR

**Figure 4:**
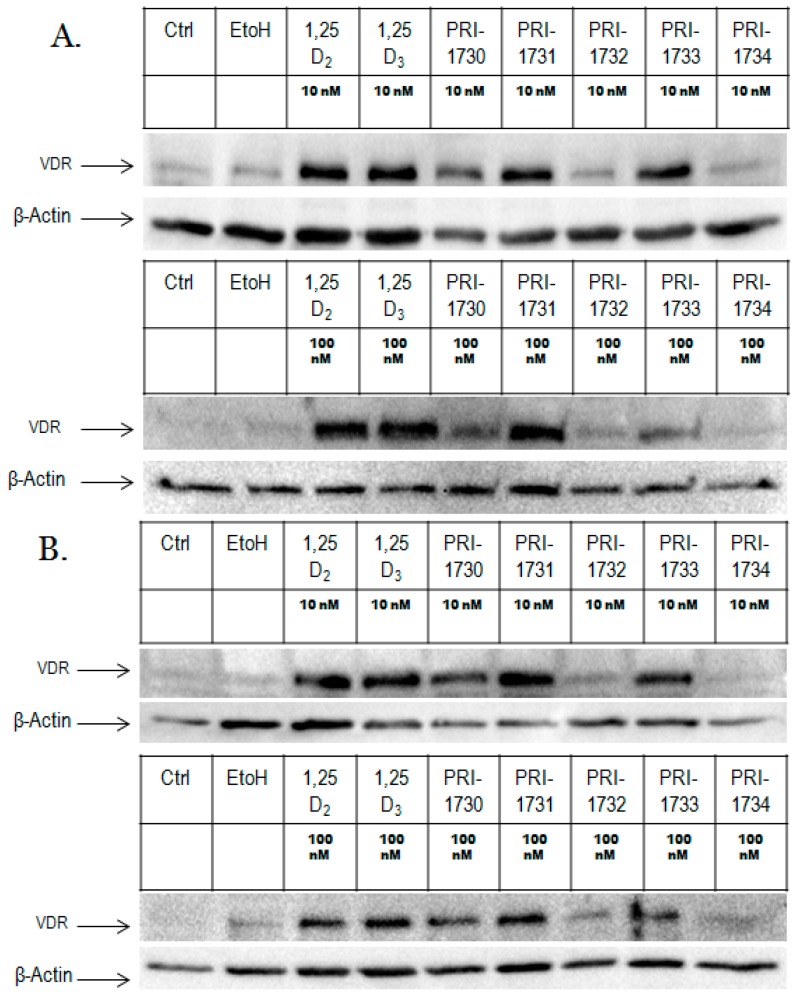
Nuclear localization of VDR protein in HL60 exposed to 1,25D_2_, 1,25D_3_ or analogues. HL60 cells were exposed to compounds at 10 nM and 100 nM concentration for 24 h (**A**) and 72 h (**B**) and then expression of VDR was determined in the nuclear fractions.

We analyzed VDR levels in the nuclear fractions of HL60 cells exposed to analogues at either 10 or 100 nM and at various time points. For these experiments, actin was used as a control. 1,25D_2_ and 1,25D_3_ induced a significant increase in VDR levels at both 10 and 100 nM following 24 h treatment. PRI-1731 and PRI-1733 elevated VDR at similar levels at both 10 nM and 100 nM, but to much lower levels than provoked by 1,25D_2_ or 1,25D_3_ by 24 h. PRI-1733 and PRI-1734 did not appear to elevate VDR at either concentration or at either of the 24 and 72 h time points. The amount of VDR appears to correlate with the overall differentiation effect (Figure 4).

### 2.3. Activation of C/EBPβ by 1,25D_2_, 1,25D_3_ and Analogues

**Figure 5:**
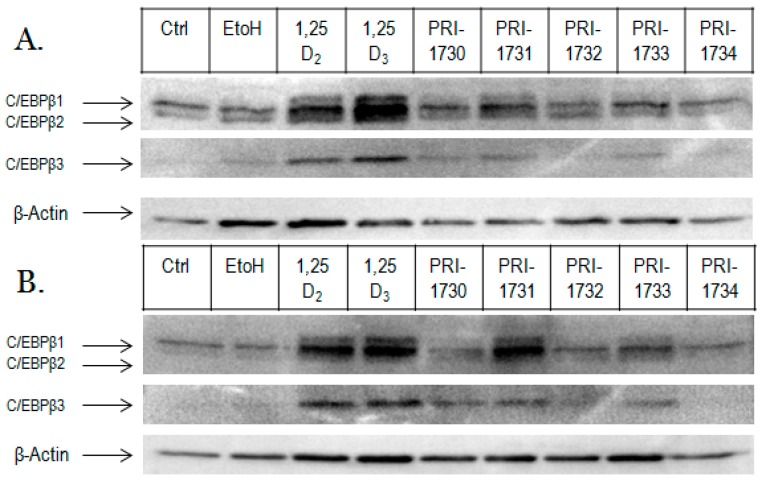
C/EBPβ isoforms in HL60 cells treated with 1,25D_2_, 1,25D_3_ and analogues at 10 nM (**A**) and 100 nM (**B**) concentrations. HL60 cells were treated for 72 h with 1,25D_2_, 1,25D_3_ and analogues. The nuclear fraction was separated by electrophoresis and transferred onto PVDF membrane and probed with antibodies against C/EBPβ, and β-actin as fractionation/loading controls. In addition to the three C/EBPβ isoforms, unidentified bands, possibly cleavage products of C/EBPβ, are present.

We also determined the ability of each analogue to upregulate expression of C/EBPβ protein. Here, we determined C/EBPβ levels in the nucleus of HL60 cells following 72 h treatment with 10 or 100 nM 1,25D_2_, 1,25D_3_ and analogues. Both 1,25D_2_ and 1,25D_3_ strongly upregulated C/EBPβ2 and C/EBPβ3 at both 10 and 100 nM (Figure 5).

### 2.4. Binding Affinity of Analogues to VDR

Next, the affinities of the analogues to VDR were assessed using a fluorescence polarization (FP)-based competition assay. The affinity of analogues for VDR was checked using a wide range of concentrations and compared to that of 1,25D_2_ and 1,25D_3_. Dose-response curves were plotted, and IC_50_ values were calculated from these dose-response curves (Table 1).

**Table 1:** The vitamin D receptor (VDR) binding affinity expressed as IC_50_ and percentage activity.

	1,25D_3_	1,25D_2_	PRI-1730	PRI-1731	PRI-1732	PRI-1733	PRI-1734
IC_50_	4.494 × 10^−9^	1.466 × 10^−8^	2.006 × 10^−7^	2.218 × 10^−8^	2.138 × 10^−7^	4.386 × 10^−6^	2.641 × 10^−6^
RBA ^a^	100	30.66	2.175	20.26	2.03	0.102	0.170

^a^ The potency of 1,25D_3_ is normalised to 100; RBA: relative binding affinity.

Binding affinities for the analogues were shown to be lower than that of 1,25D_3_ and 1,25D_2_, with analogue PRI-1731, the most biologically active of these analogues, displaying the highest affinity to VDR. We have previously noted that there appears to be no correlation with differentiation abilities and affinity to the VDR [10].

### 2.5. Metabolic Resistance of Analogs to CYP24A1

**Figure 6:**
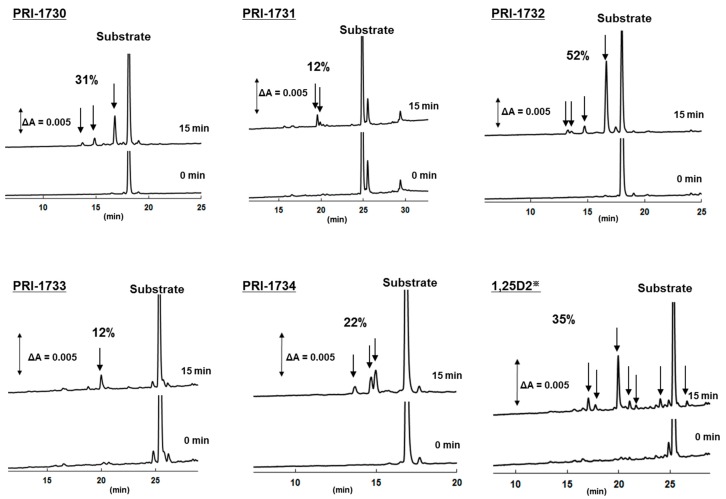
HPLC profiles of 1,25D_2_ and of its analogues and their metabolites by human CYP24A1. The peaks with arrows indicate putative metabolites. The HPLC profile of 1,25D_3_ is shown in Figure S1 in supplementary information of this paper.^※^ The metabolic profile of native 1,25D_2_ by human CYP24A1 was nearly the same as in our previous reports [15,16]. ∆A indicates the absorbance difference at 265 nm.

The catabolism of each analogue by human CYP24A1 was analyzed using the membrane fraction prepared from the recombinant *Escherichia coli* cells expressing hCYP24A1 [15,16]. Figure 6 shows the HPLC profile of each analogue and the metabolites from catabolism by hCYP24A1. Table 2 shows the conversion ratio of each substrate into the metabolites. The metabolic conversion of analogue PRI-1731 was only 12%, which was much lower than that of native 1,25D_2_, 35%. The only structural difference between PRI-1731 and 1,25D_2_ is the altered geometry of the triene system *i.e.*, a 5,6-*trans* instead of 5,6-*cis*. This finding suggests that 5,6-*trans* modification is contributing substantially to the increased stability of the analogue in a series of analogues with the side-chain of the regular length. Unexpectedly, reversing the chirality at C-24 in the 5,6-*trans* analogue PRI-1731 from the natural (24*S*) into the (24*R*) configuration (PRI-1733) did not have any effect on metabolic conversion, which remained equal to 12%, while it did for functional activity of other 1,25D_2_ analogues (PRI-1732 and PRI-1734). In a series of 5,6-*trans* analogues of 1,25D_2_ with the natural configuration at C-24, the addition of C-22 hydroxyl with saturated C22-C23 bond resulted in a dramatic loss of metabolic resistance from 12% for PRI-1731 to 52% for its 22-hydroxy derivative PRI-1732. We suspect that high loss of metabolic resistance of PRI-1732 may be due to the electronic and steric effects of interaction of this analogue with the hydroxylating enzyme. Preparation, isolation and structure identification of the major metabolite of PRI-1732 by HPLC/MS/MS is currently underway in our laboratories. The decrease of metabolic resistance was also observed from 12% for PRI-1733 to 22% for PRI-1734. Addition of C-22 hydroxyl is not that influential for natural 5,6-*cis* vitamins, as the conversion of 22-hydroxy analogue PRI-1730 is 31% as compared to 35% for parent 1,25D_2_. For C-22 hydroxy analogues PRI-1730 and PRI-1732, 5,6-*trans* modification decreased metabolic resistance. Interestingly, consistently in our studies [17], 1,25D_2_ is more stable than 1,25D_3_ and, therefore, 1,25D_2_ analogues should be preferred in this respect (Table 2).

**Table 2:** Metabolic conversion of active forms of vitamin D and its analogues by human CYP24A1 (%).

Compound	1,25D_3_	1,25D_2_	PRI-1730	PRI-1731	PRI-1732	PRI-1733	PRI-1734
Metabolic conversion (%)	44	35	31	12	52	12	22

Data represent means of at least 3 independent experiments.

### 2.6. Identifying If PRI-1734 Has Antagonistic Activity

**Figure 7:**
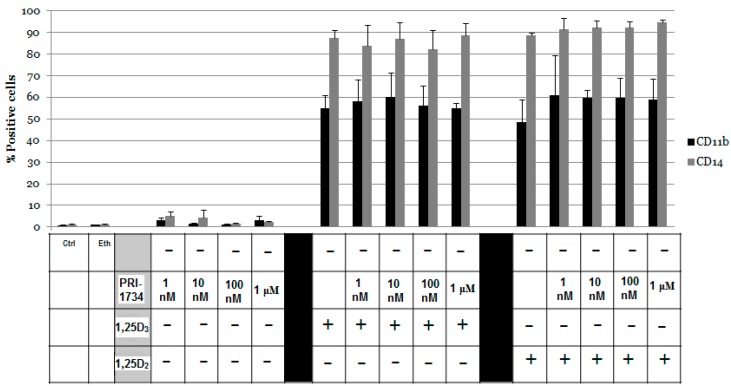
Effect of PRI-1734 in combination with 1,25D_3_ or 1,25D_2_ on differentiation of HL60 cells. HL60 cells were exposed to 10 nM of either 1,25D_2_ or 1,25D_3_ in combination with PRI-1734 at different concentrations of: 1 nM, 10 nM, 100 nM, and 1 μM. The expression levels of both CD11b and CD14 were studied. Percentages of positive cells are presented in the *y*-axis. Ctrl indicates untreated control cells, Eth indicates ethanol (vehicle) treated control cells.

Given that PRI-1734 appeared to be inactive in promoting differentiation in HL60 cells, we hypothesized that PRI-1734 may have properties antagonistic to 1,25D_2_ and 1,25D_3_. Thus, HL60 cells were exposed to PRI-1734 at increasing concentrations in combination with either 10 nM 1,25D_2_ or 10 nM 1,25D_3_. Results presented in Figure 7 show that PRI-1734 does not appear to have antagonistic properties, as the differentiation induced by both 1,25D_2_ and 1,25D_3_ remained unaffected.

## 3. Discussion

To enhance the therapeutic properties of 1,25D_3_, it is necessary to obtain analogues that have increased benefits to risk ratios, as compared to the parent compound. Thousands of different analogues have been synthesized in the past, but the optimal structure-function relationship has yet to be defined [18]. In previous research, we documented that analogues in which the side-chain has unsaturation at C-22 and methyl at C-24, and which is extended and branched with methyl or ethyl groups have increased differentiation-inducing properties [8,9]. Addition of 19-*nor* modification has led to reduced calcaemic activities and general toxicity for mice, and an even stronger differentiation-inducing potential [10].

Here we have demonstrated for the first time that introducing the 24-*epi* modification into the structure of 22-hydroxy-5,6-*trans* analogue (PRI-1732) resulted in a complete lack of differentiation-inducing activity of the compound. The inactive analogue PRI-1734 was a good substrate for CYP24A1 and was oxidized by this enzyme into the three more polar metabolites in a total of 22% comparing to 35% for 1,25D_2_ and 52% for the parent analogue PRI-1732.

Previous research has shown that the overall biological activity of a given analogue depends on interplay between various properties. For example, affinity of the analogue to VDR rarely correlates with differentiation-inducing potential [18]. The speed of catabolism also cannot explain all the differences in the activities of analogues [17]. Our studies have shown that the ability of a given analogue to induce nuclear accumulation of VDR and C/EBPβ transcription factors in AML cells is the best predictor of differentiation-inducing properties [10,19,20].

## 4. Materials and Methods

### 4.1. Chemicals and Antibodies

1,25D_3_, 1,25D_2_ and analogues were manufactured at the Pharmaceutical Research Institute (Warsaw, Poland). The compounds were placed in glass ampoules at and kept −20 °C. Analogues were dissolved in absolute ethanol at 100 μM and further diluted in culture medium for each required experimental concentration. Antibodies for flow cytometry CD14-PE and CD11b-FITC were from ImmunoTools (Friesoythe, Germany). Antibodies for Western blotting and chemiluminescence blotting substrate were from Santa Cruz Biotechnology Inc. (Santa Cruz, CA, USA).

### 4.2. Cell Lines

HL60 cells were from the Institute of Immunology and Experimental Therapy in Wroclaw, Poland. The cells were cultured in RPMI 1640 medium containing 10% fetal calf serum (FCS, Sigma, St. Louis, MO, USA), 100 units/mL penicillin and 100 μg/mL streptomycin (Sigma). The cells were cultured at standard cell culture conditions, *i.e.*, humidified atmosphere of 95% air and 5% CO_2_ at 37 °C. The cell number and viability were determined by haemocytometer counts and trypan blue (0.4%) exclusion. For all experiments, the cells were suspended in fresh medium containing 1,25D_2_, 1,25D_3_, analogue or the equivalent volume of ethanol as a vehicle control.

### 4.3. Determination of Cell Differentiation by Flow Cytometry

The expression of cell surface markers of monocytic differentiation was determined by flow cytometry. The cells were treated with compounds at various concentrations for 96 h and then stained with CD11b and CD14 antibodies. Cells were washed twice in 500 μL PBS and incubated for 1 h on ice with 1 μL CD14-PE and 1 μL CDllb-FITC. Cells were washed three times with PBS containing 0.1% BSA and suspended in 400 μL PBS prior to analysis on the Becton Dickinson Accuri C6 (San Jose, CA, USA). Data analysis was performed using Becton Dickinson Accuri C6 software.

### 4.4. Preparation of Cell Lysates

Cells (5 × 10^6^) were washed three times with PBS and lysed for 20 min on ice in 80 μL of lysis buffer pH 7.5 (20 mM Tris, 1% Triton X-100,150 mM NaCl, 1 mM EDTA, 1 mM EGTA) with added protease inhibitor cocktail (Roche Diagnostics, Mannheim, Germany). The lysates were centrifuged for 5 min, at 18,000× *g*, at 4 °C in order to separate them. Supernatants were designated cytoplasmic (C) fractions and the nuclei remaining in pellets were washed and sonicated for 5 s in the same lysis buffer as before. In order to obtain the nucler (N) fraction, samples were centrifuged for 5 min at 18,000× *g*, at 4 °C. Twenty microliters of 5× SDS sample buffer were added to each fraction and boiled for 10 min in order to denature the proteins.

### 4.5. Western Blotting

Twelve-percent SDS-PAGE gels were used to separate 30 μL of cell lysates (derived from 5 × 10^6^ cells) and transferred to the PVDF membranes. The membranes were incubated with primary antibody (2 h), followed by horseradish peroxidise-conjugates secondary antibody (1 h). Chemiluminescence substrate was used to identify protein bands. Membranes were stripped, and reprobed with additional antibodies. The loading control for all experiments was actin. Membranes were scanned and bands quantified using Image J 1.34s software (freeware by Wayne Rasband, NIH).

### 4.6. Human VDR Binding Assay

Binding affinity to VDR was evaluated using a Polarscreen Vitamin D receptor competitor assay, under manufacturer conditions (Life Technologies, Carlsbad, CA, USA). The polarised fluorescence was measured using Envision (Perkin-Elmer, Waltham, MA, USA). All compounds were evaluated within the range 10^–11^ to 10^–5^ M, half maximal inhibitory concentration (IC_50_) values were calculated using the average of measured values.

### 4.7. Metabolic Resistance of Analogues to CYP24A1

Metabolism of each analogue by recombinant human CYP24A1 was examined using the reconstituted system containing the membrane fraction prepared from the recombinant , *Escherichia coli* cells expressing human CYP24A1 [15,16]. The reaction mixture containing 2.0 μM bovine adrenodoxin (ADX), 0.2 μM bovine adrenodoxin reductase (ADR), 20 nM CYP24A1, 5 µM each analogue, 1 mM NADPH, 100 mM Tris-HCl (pH 7.4), and 1 mM EDTA was incubated for 15 min at 37 °C. Four-volumes of chloroform/methanol (3:1) were used to terminate each reaction in combination with vigorous shaking. Following this, the organic phase was retrieved and dried. The resultant residue was dissolved in acetonitrile, and was centrifuged at 20,000× *g* for 15 min. The resultant supernatant was submitted to High Performance Liquid Chromatography (HPLC) according to the conditions described here: column, YMC-pack ODS-AM (4.6 mm × 300 mm) (YMC Co., Tokyo, Japan); UV detection, 265 nm; flow late, 1.0 mL/min; column temperature, 40 °C; linear gradients of 20%–100% acetonitrile aqueous solution per 25 min followed by 100% acetonitrile for 5 min.

## 5. Conclusions

Data presented here document that the modifications introduced to our new double point modified 1,25D_2_ analogues have not increased their differentiation-inducing properties. Nevertheless, the modifications introduced resulted in a very divergent group of analogs having moderate to completely abolished cell differentiation activity. These analogues had affinities to VDR similar or lower than 1,25D_2_, and were less efficient in inducing differentiation of AML cells. Based on flow cytometry assays, we can order their differentiation-inducing activities as follows: 1,25D_3_ > 1,25D_2_ > PRI-1731 > PRI-7130 ≈ PRI-1733 > PRI-1732 > PRI-1734. They induced nuclear accumulation of VDR and C/EBPβ less efficiently than 1,25D_3_ and 1,25D_2_. Since the analogues PRI-1732 and PRI-1734 were the least active of all tested compounds, we hypothesize that the combination of a 22-hydroxyl with 5,6-*trans* modification has led either to decreased transport of the analogues into the cells or to a diminished interaction with VDR inside the cells.

## Supplementary Materials

Supplementary materials can be found at http://www.mdpi.com/1422-0067/17/2/91/s1.
