# Supplementary material for: Biological Evaluation of Double Point Modified Analogues of 1,25-Dihydroxyvitamin D2 as Potential Anti-Leukemic Agents
Source: Int J Mol Sci. 2016 Feb 1;17(2):91. doi: 10.3390/ijms17020091 (PMC4783874; doi:10.3390/ijms17020091)
Supplement: Supplementary file 1 [file ijms-17-00091-s001.pdf]

Supplementary Materials: Biological Evaluation of Double Point Modified Analogues of 1,25-Dihydroxyvitamin D_2 as Potential Anti-Leukemic Agents

Aoife Corcoran, Sharmin Nadkarni, Kaori Yasuda, Toshiyuki Sakaki, Geoffrey Brown, Andrzej Kutner and Ewa Marcinkowska

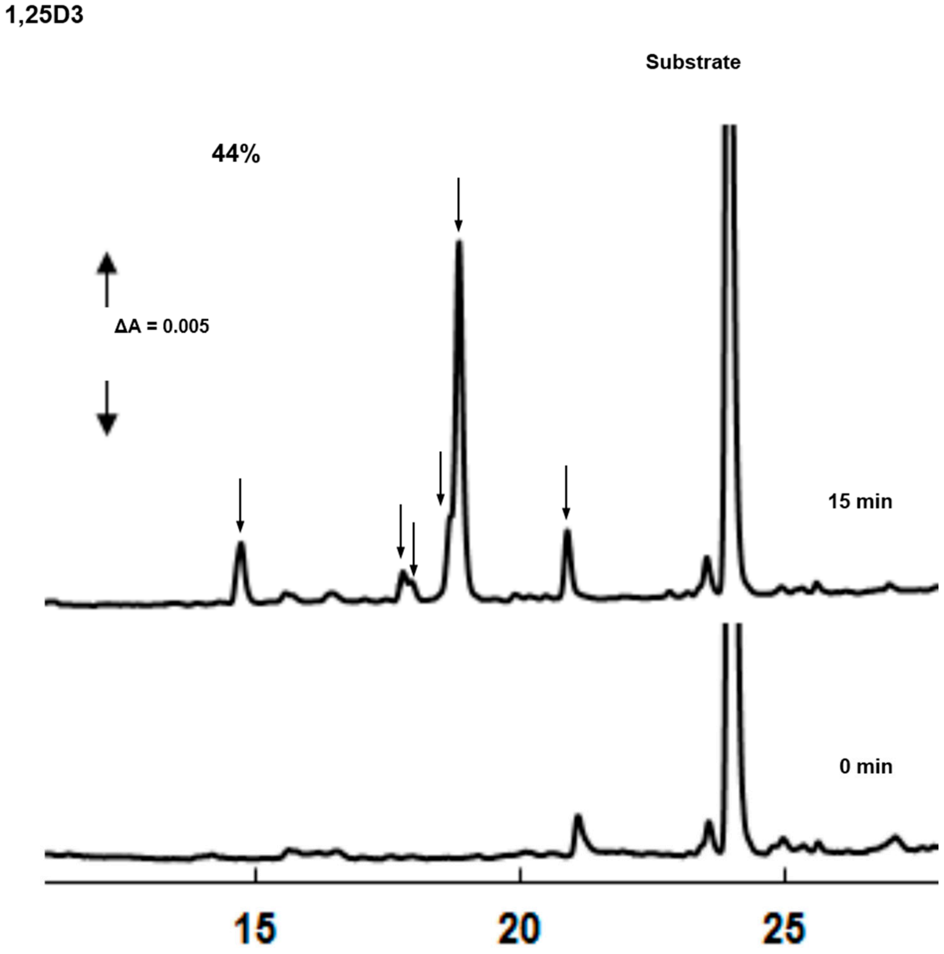

Figure S1. HPLC profiles of 1,25D_3 and its metabolites by human CYP24A1. The peaks with arrows indicate putative metabolites. The metabolic profile was nearly the same as that in our previous reports [15,17].
